# Rapid Accumulation of CD14^+^CD11c^+^ Dendritic Cells in Gut Mucosa of Celiac Disease after *in vivo* Gluten Challenge

**DOI:** 10.1371/journal.pone.0033556

**Published:** 2012-03-16

**Authors:** Ann-Christin Røberg Beitnes, Melinda Ráki, Margit Brottveit, Knut Erik Aslaksen Lundin, Frode Lars Jahnsen, Ludvig Magne Sollid

**Affiliations:** 1 Centre for Immune Regulation and Department of Immunology, Oslo University Hospital – Rikshospitalet, Oslo, Norway; 2 Department of Medicine, Oslo University Hospital – Ullevål and University of Oslo, Oslo, Norway; 3 Department of Medicine, Oslo University Hospital – Rikshospitalet, Oslo, Norway; 4 Centre for Immune Regulation and Department of Pathology, Oslo University Hospital – Rikshospitalet and University of Oslo, Oslo, Norway; 5 Centre for Immune Regulation and Department of Immunology, University of Oslo, Oslo, Norway; Tulane University, United States of America

## Abstract

**Background:**

Of antigen-presenting cells (APCs) expressing HLA-DQ molecules in the celiac disease (CD) lesion, CD11c^+^ dendritic cells (DCs) co-expressing the monocyte marker CD14 are increased, whereas other DC subsets (CD1c^+^ or CD103^+^) and CD163^+^CD11c^−^ macrophages are all decreased. It is unclear whether these changes result from chronic inflammation or whether they represent early events in the gluten response. We have addressed this in a model of *in vivo* gluten challenge.

**Methods:**

Treated HLA-DQ2^+^ CD patients (n = 12) and HLA-DQ2^+^ gluten-sensitive control subjects (n = 12) on a gluten-free diet (GFD) were orally challenged with gluten for three days. Duodenal biopsies obtained before and after gluten challenge were subjected to immunohistochemistry. Single cell digests of duodenal biopsies from healthy controls (n = 4), treated CD (n = 3) and untreated CD (n = 3) patients were analyzed by flow cytometry.

**Results:**

In treated CD patients, the gluten challenge increased the density of CD14^+^CD11c^+^ DCs, whereas the density of CD103^+^CD11c^+^ DCs and CD163^+^CD11c^−^ macrophages decreased, and the density of CD1c^+^CD11c^+^ DCs remained unchanged. Most CD14^+^CD11c^+^ DCs co-expressed CCR2. The density of neutrophils also increased in the challenged mucosa, but in most patients no architectural changes or increase of CD3^+^ intraepithelial lymphocytes (IELs) were found. In control tissue no significant changes were observed.

**Conclusions:**

Rapid accumulation of CD14^+^CD11c^+^ DCs is specific to CD and precedes changes in mucosal architecture, indicating that this DC subset may be directly involved in the immunopathology of the disease. The expression of CCR2 and CD14 on the accumulating CD11c^+^ DCs indicates that these cells are newly recruited monocytes.

## Introduction

Celiac disease (CD) is a chronic small intestinal inflammatory condition caused by an inappropriate immune response to gluten proteins of wheat, rye and barley. The condition is common with a prevalence of about 1%. There is a strong association of CD with certain HLA alleles. The majority of patients carry HLA-DQ2.5 (80–95%) whereas most of the remaining patients carry HLA-DQ8 [1], [2]. The histopathology of CD is characterized by villous blunting, crypt hyperplasia and increased number of CD3^+^ intraepithelial lymphocytes (IELs) [3]. The current treatment for CD is lifelong gluten-free diet (GFD).

Gluten-reactive T cells in the gut appear to play a central role in the immunopathogenesis of CD, but how and where these T cells get activated by interaction with antigen-presenting cells (APCs) fronting them with gluten antigen are not well understood. The priming of naïve T cells is likely to take place in organized lymphoid tissue whereas activation of effector T cells probably takes place in the gut mucosa. In a previous study we found that duodenal lamina propria HLA-DQ^+^ APCs could be divided into CD11c^+^ dendritic cells (DCs) and CD68^+^CD11c^−^ macrophages and that gluten challenged CD11c^+^ DCs isolated from celiac lesions more efficiently activated gluten-reactive T cells than their macrophage counterparts [4]. More recently we further subclassified the CD11c^+^ DC population into cells expressing either CD103, CD1c or CD163. Co-staining experiments showed that CD68^+^ and CD163^+^ cells were completely overlapping populations. CD103^+^ and CD1c^+^ DCs were partly overlapping populations, whereas the majority of CD11c^+^CD163^+^ DCs co-expressed the monocyte marker CD14, suggesting that they were derived from monocytes. Furthermore, we observed that the density of CD14^+^CD11c^+^ DCs was increased while the density of CD103^+^ DCs, CD1c^+^ DCs and CD163^+^CD11c^−^ macrophages was decreased in the active celiac lesion [5]. The differences between untreated CD patients and healthy controls with respect to mucosal APC populations suggest dynamic regulation of these populations, but little is known about the kinetics of these changes in relation to gluten exposure.

To address this issue we used a unique *in vivo* model in which the density of mucosal APC subsets of treated CD patients and gluten-sensitive control subjects where determined before and after a three-day gluten challenge. We show that there are rapid dynamic changes in the APC populations in the challenged duodenal mucosa of CD patients, but not in gluten-sensitive controls.

## Materials and Methods

### Ethics statement

The study was approved by the Regional Committee for Medical Research Ethics in South-East Norway and the Privacy Ombudsman for Research at Oslo University Hospital – Rikshospitalet (Oslo, Norway), and it is registered at http://clinicaltrials.gov/ct2/results?term=NCT01100099. The study complies with the Declaration of Helsinki. All the participants gave their written informed consent.

### Subjects

We studied CD patients (n = 12; mean age 51 years, range 37–65, 7 females) and gluten-sensitive control subjects (n = 12; mean age 45 years, range 29–65, 12 females) who all were HLA-DQ2^+^. The subjects have been described in detail elsewhere [6]. Twelve CD patients, diagnosed on the basis of typical histopathological changes in duodenal mucosa [7] and from whom we had cryopreserved biopsies available, as well as twelve randomly selected gluten-sensitive control subjects were included. Both the CD patients and the gluten-sensitive control subjects had been on a strict GFD for at least 4 weeks prior to the study. The gluten-sensitive participants had initiated their GFD without being examined for CD by gastroendoscopy. Three had negative IgA TG2 serology before commencing the GFD and nine had unknown serology. Most of these subjects had experienced symptoms, like abdominal discomfort and/or diarrhea, on a gluten containing diet, and the symptoms improved on dietary gluten elimination. All participants were challenged orally with four slices (∼160 g) of gluten-containing white bread every day for three days. Duodenal biopsy specimens were obtained before challenge and at day four. During the gluten challenge, symptoms like bloating, diarrhea, constipation and satiety were observed in six of twelve CD patients and in seven of twelve gluten-sensitive control subjects. There were only four CD patients who experienced histological changes after challenge; two patients changed from Marsh 1 to 3a, one patient changed from Marsh 2 to 3a, and one patient changed from Marsh 0 to 3b. One patient was unchanged Marsh 3a, whereas the seven other patients were unchanged Marsh 0. Thus, there were no statistically significant changes in Marsh grade after a three-day gluten challenge amongst the CD patients. Biopsies from gluten-sensitive control subjects were scored as Marsh 0 both before and after challenge in all cases. The gluten-sensitive subjects were included as controls because they adhered to a GFD and were willing to undergo a gluten challenge. These subjects are highly unlikely to suffer from CD as they did not react with appearances of HLA-DQ2-gluten tetramer positive CD4^+^ T cells in the peripheral blood after the gluten challenge [6].

In addition to the material obtained from individuals of the challenge study, duodenal biopsies were obtained from three treated CD and three untreated CD patients as well as from four patients with normal histology who were examined with gastroendoscopy as part of the routine diagnostic workup. Blood samples were also obtained from two treated CD patients who were not challenged with gluten. These subjects were included due to limited material available from the participants of the challenge study. Finally, CD14^+^ monocytes were isolated from buffy coats obtained from two HLA-DQ2^+^ healthy individuals.

### Multicolor immunofluorescence staining

Two biopsy specimens from each subject were oriented on thin slices of carrot, embedded in Tissue Tek optimal cutting temperature (O.C.T.) compound, snap frozen “bed-side” in liquid nitrogen and stored at −70°C. H+E stained sections of both specimens were evaluated and cryosections of the best oriented tissue sample were cut in series at 4 µm and dried in room temperature (RT) over night. The sections were then fixed with acetone for 10 minutes, dried for 15 minutes, wrapped in aluminium foil and stored at −20°C until use. Two- or three-color immunofluorescence staining was performed. To determine the density and phenotype of HLA-DQ^+^ APCs, cryosections were first incubated with the following combinations of mouse monoclonal antibodies (mAbs) for 1 hour at RT: anti-HLA-DQ (clone SPV-L3, IgG2a, 1.3 µg/mL, kind gift from H. Spits, Amsterdam, Netherlands [8]) with anti-CD163 (clone RM3/1, IgG1, 10 µg/mL, lot 818176, abcam, Cambridge, UK); anti-CD11c (clone CRB-p150/4G1, IgG2a, 5 µg/mL; Biosource, Camarillo, CA) with either anti-CD103 (clone Ber-ACT8, IgG1, 1/200, kind gift from H. Dürkop, Berlin, Germany [9]), CD1c (clone M241, IgG1, 5 µg/mL, Ancell Corporation, Bayport, MN), CD14 (clone 18D11, IgG1, 0.3 µg/mL, kind gift from T. Espevik, Trondheim, Norway [10]) or CD163 (clone RM3/1). Polyclonal antibody specific for cytokeratin (rabbit anti-human IgG, 1/100, H. Huitfeldt, Oslo, Norway [11]) was added to the primary mAb mixtures to visualize the epithelium. The sections were briefly rinsed with phosphate-buffered saline (PBS) and then incubated with biotinylated goat anti-mouse IgG2a (2.5 µg/mL; SouthernBiotech, Birmingham, AL) for 1.5 hour, followed by a combination of Cy2-labeled streptavidin (1 µg/mL, Amersham Biosciences, Buckinghamshire, UK) and Cy3-labeled goat anti-mouse IgG1 (2.9 µg/mL; SouthernBiotech) for 30 minutes. Initial testing showed that the staining intensity using anti-CD103 was enhanced by prefixing the sections with paraformaldehyde-lysine-periodate (PLP) for 10 minutes. In that case, incubation with anti-CD103 and anti-CD11c was followed by a combination of FITC-labeled goat anti-mouse IgG2a (20 µg/mL; SouthernBiotech) and Cy3-labeled goat anti-mouse IgG1 for 30 minutes. Before mounting, the sections were washed in Hoechst 33258, pentahydrate (bis-benzimide) (1 µg/mL; Invitrogen, Paisley, UK) for 5 minutes to visualize cell nuclei. When anti-cytokeratin was included, 7-amino-4-methylcoumarin-3-acetic acid (AMCA)-labeled goat anti-rabbit IgG (1/10–1/20; Vector Laboratories, Burlingame, CA) was added in the final step. Irrelevant isotype- and concentration-matched primary mAbs were used as negative control in all experiments.

### Immunoenzyme staining

Four biopsies from each subject were formalin-fixed and paraffin-embedded in the same paraffin block. Sections were cut in series at 4 µm and dewaxed. Heat-induced epitope retrieval (HIER) was performed by boiling sections for 20 minutes in Tris-EDTA (pH = 9) in a water bath followed by cooling for 20 minutes at RT. Immunoenzyme staining was performed with Ventana Ultra View DAB Detection Kit (Ventana Medical Systems, Inc., Tucson, AZ). Sections were pre-blocked with H_2_O_2_ for 8 minutes. Primary antibodies used were either monoclonal rabbit anti-human CD3 (clone SP7, IgG, 1/100, Thermo Scientific, Fremont, CA) or monoclonal mouse anti-human neutrophil elastase (clone NP57, IgG1, 1/600, Dako) for 30 minutes in RT. When stained with anti-neutrophil elastase (clone NP57) sections were not pretreated with HIER. Antibody Diluent (S0809; Dako) replaced the primary antibody as negative control. All sections were counterstained with haematoxylin.

### Evaluation of tissue staining results

Examination of immunofluorescence stainings was performed with an epifluorescence microscope (Nikon Eclipse 80i, Nikon Corporation, Tokyo, Japan). To determine the density of different cell populations, all immunostained cells in lamina propria were counted to a depth of ∼0.5 mm from the basolateral side of the surface epithelium. The area of lamina propria was estimated by superimposing a grid (10×10 lines; 0.242×0.242 mm) parallel to the muscularis mucosa. On average, 7 grids were examined for every section. Combined fluorescent microscopy and differential interference contrast microscopy (DIC) was used to visualize eosinophils as previously described [12].

Immunoenzyme-stained formalin-fixed sections were examined by light microscopy (Nikon Eclipse 50i, Nikon Corporation). To determine the density of IELs, all intraepithelial CD3^+^ cells in the upper half of the villi with satisfactory morphology were counted, and the density was given as IELs per 100 epithelial cells (EPCs). The density of neutrophils in lamina propria was determined by superimposing a grid parallel to the muscularis mucosa as described above.

All sections were examined at 400 X magnification by the same investigator (A-C.R. Beitnes), blinded to patient identity and diagnosis.

### Multicolor flow cytometry

Multiple biopsies (4–10) were collected in ice-chilled RPMI medium and processed further in the laboratory within 30 minutes. Preparations of single cell suspensions were performed as follows: EPCs and IELs were removed by incubation with 2 mM EDTA in PBS twice for 30 minutes with continuous rotation at 37°C. Single-cell suspensions were obtained by digesting the remaining material with 1 mg/mL Blend Collagenase (C-8051; Sigma, St. Louis, MO) for 60 minutes with rotation at 37°C. The cell suspension was then filtered through a 40 µm cell strainer and washed with PBS. For analysis of blood cells, peripheral blood mononuclear cells were isolated from acid-citrate-dextrose (ACD) blood using standard protocols for density centrifugation with Lymphoprep. Cells were transferred onto V-bottomed 96-well plates, washed in PBS containing 0.5 mM ethylenediaminetetraacetic acid (EDTA) and 3% foetal calf serum (FCS) and stained with directly labeled antibodies on ice for 30 minutes. After incubation, cells were briefly washed, resuspended in PBS with 3% FCS and analyzed with a LSRII (BD Biosciences, Franklin Lakes, NJ) instrument. To exclude dead cells, 0.2 µg/mL propidium iodide (PI) was added to the samples immediately prior to analysis. 2−4×10^5^ cells were analyzed in each sample.

The following antibodies and dilutions were used: anti-CD45-FITC, -PE, -APC and -Pacific Blue (all clone HI30, 1/20) and anti-HLA-DR-eFluor450 (clone L243, 1/40) from eBioscience, San Diego, CA; anti-CD11c-Alexa488 (clone 3.9, 1/20), anti-CD14-APC-Cy7 (clone HCD14, 1/20), anti-CCR2-Alexa 647 (clone TG5/CCR2, 1/20) and IgG2b-Alexa647 isotype control (clone MPC11, 1/20) from Biolegend, San Diego, CA; anti-CD11c-PE (clone S-HCL-3, 1/15), anti-HLA-DR-PE-Cy7 (clone L243, 1/100) from BD Biosciences and anti-DC-SIGN/CD209-PE (clone DCN46, 1/15) from BD Pharmingen, San Diego, CA.

### T cell assay

Monocytes were isolated from peripheral blood mononuclear cells using MACS CD14 MicroBeads (Miltenyi Biotec STED) according to the manufacturer's instructions. Recovered cells were stained for CD14 and CD11c to assess purity. Monocytes (2×10^4^ cell) were transferred into 96-well plates and incubated overnight with or without peptide antigen and with or without recombinant IFN-γ (100 U/ml; R&DSystems, Minneapolis, MN). Two gliadin peptides harboring the HLA-DQ2.5-glia-α2 epitope were tested; PQPELPYPQPQL (DQ2-α2) at 10 µM or LQLQPFPQPELPYPQPELPYPQPELPYPQPQPF (33-mer) at 2 µM [13], [14]. The CD14^+^ monocytes were then washed and incubated with 50.000 cells of the gut derived T-cell clone TCC493.3.4.5 specific for the HLA-DQ2.5-glia-α2 epitope. The gluten-reactive T-cell clone was generated as previously described [15]. T-cell proliferation was evaluated after 72 hours by uptake of [^3^H]thymidine (1 µCi/well (0.037 MBq/well); Hartmann Analytic, Braunshweig, Germany), which was added to the wells 24 hours before harvesting with an automated harvester (Mach III; TomTec, Hamden, CT). Incorporated radioactivity was measured by liquid scintillation counting (Wallac MicroBeta TriLux 1450; PerkinElmer, Wellesley, MA). The assays were performed in triplicates.

### Statistical analysis

Wilcoxon matched pairs test was used to compare the density of different cell populations in the duodenal mucosa before and after gluten challenge. GraphPad Prism 4 software (GraphPad Software, La Jolla, CA) was used for statistical analysis.

## Results

### CD14^+^CD11c^+^ DCs are selectively increased after a three-day gluten challenge in CD patients

**Figure 1:**
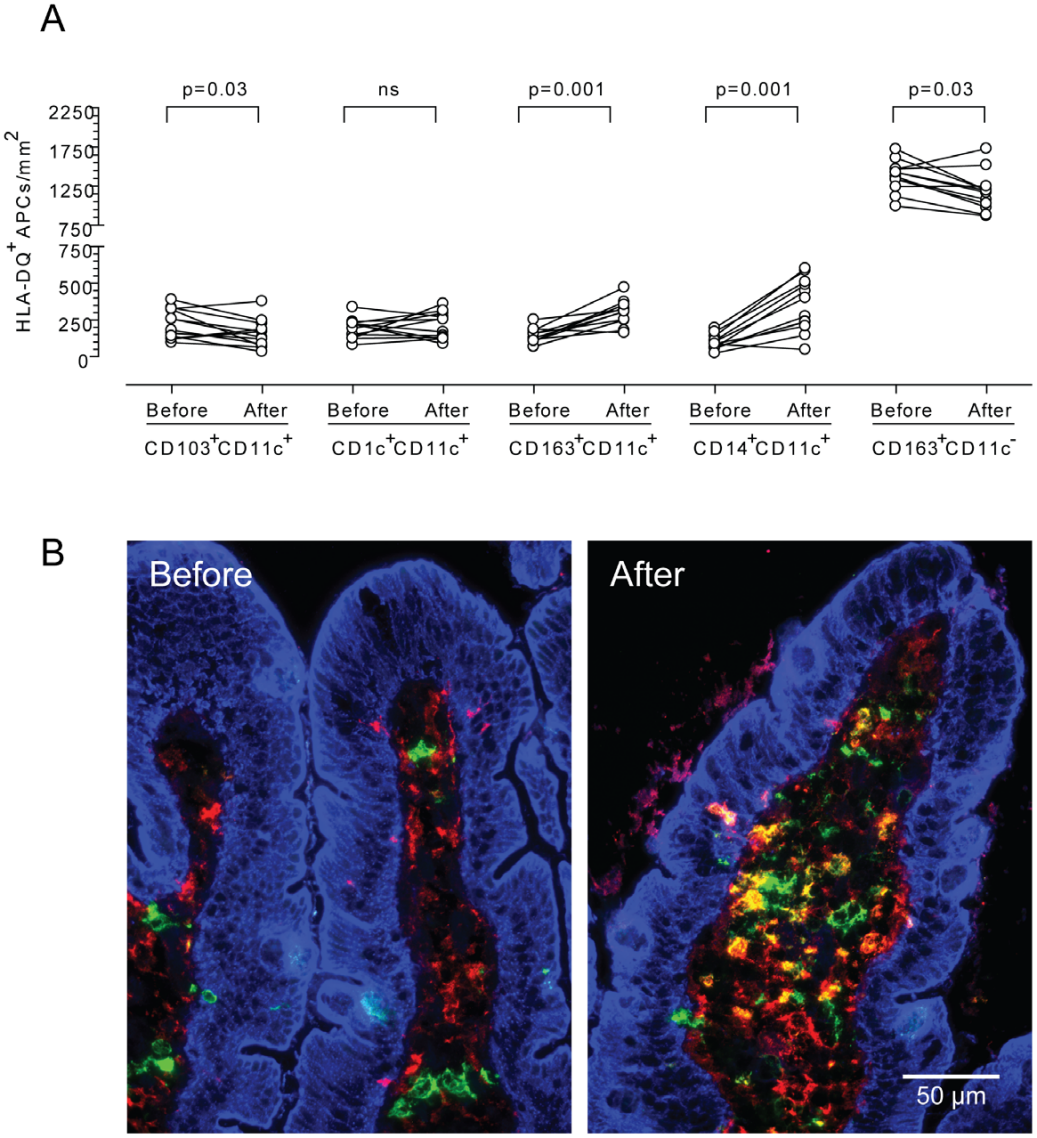
CD14^+^CD11c^+^ dendritic cells are selectively increased in duodenal mucosa of celiac disease patients after short-term gluten challenge. Density of HLA-DQ^+^ antigen-presenting cell subsets in cryosections from celiac disease (CD) patients on gluten-free diet (GFD) before and after a three-day gluten challenge. The density of CD163^+^CD11c^−^ macrophages is calculated by subtracting the number of CD163^+^CD11c^+^ cells from the total number of CD163^+^HLA-DQ^+^ cells. Paired data are connected by lines. ns = not significant (A). Three-color immunofluorescence staining for CD11c (green), CD14 (red) and cytokeratin (blue) in cryosection of duodenal mucosa from a CD patient on GFD before and after a three-day gluten challenge. Original magnification X 400 (B).

We found that the density of HLA-DQ^+^ APCs in lamina propria was similar before and after gluten challenge in the patients with treated CD (median 1755 cells/mm^2^, range 1398–2247 vs. median 1598, range 1417–2274, respectively). Notably, however, the relative proportions of HLA-DQ^+^ APC subsets defined by expression of the markers CD11c, CD163, CD14, CD103 and CD1c were changed. By two-color immunofluorescence staining we found an increase in the density of CD14^+^CD11c^+^ (p = 0.001) and CD163^+^CD11c^+^ (p = 0.001) DCs, and a decrease in the densities of CD103^+^ DCs (p = 0.03) and CD163^+^CD11c^−^ macrophages (p = 0.03) (Figure 1). The density of CD1c^+^ DCs was unchanged. We have recently shown that CD14^+^CD11c^+^ DCs and CD163^+^CD11c^+^ DCs are mostly overlapping populations both in the normal small intestinal mucosa and in the active CD lesion [5]. In the following experiments we therefore used the co-expression of CD14 and CD11c to identify this DC subset.

### Possible mechanisms for CD14^+^CD11c^+^ DC accumulation

**Figure 2:**
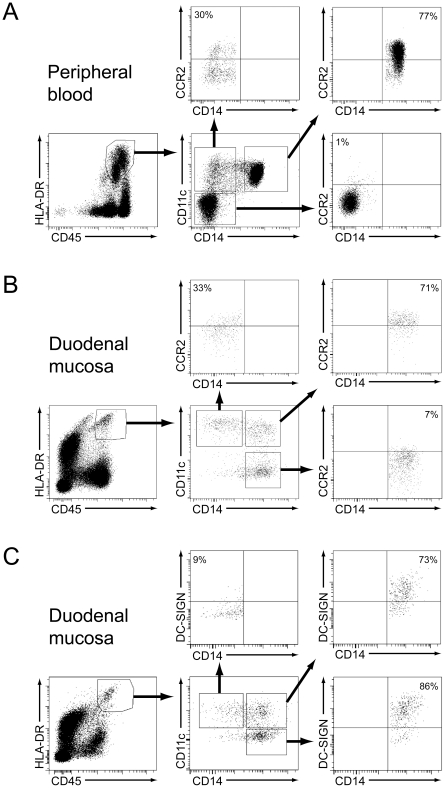
Expression of CCR2 on HLA-DR^+^ leukocytes in blood and duodenal mucosa. Flow cytometric analysis of peripheral blood mononuclear cells from treated celiac disease (CD) patient (A) and viable single cells of duodenal mucosa from treated CD patient showing the expression of CCR2 (B) and DC-SIGN (C) on CD45^+^HLA-DR^+^ cells depending on the expression of CD11c and CD14. Dead cells were excluded by adding 0.2 µg/mL propidium iodide immediately before acquisition. The data are representative for two independent experiments.

**Figure 3:**
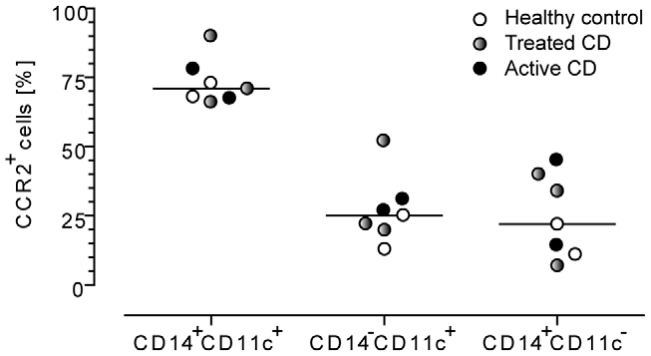
CCR2 is highly expressed on CD14^+^CD11c^+^ dendritic cells in duodenal mucosa. Percentage of CCR2^+^ cells in subsets of antigen-presenting cells from duodenal mucosa digests assessed by flow cytometry. Median is indicated by horizontal line. CD = celiac disease.

The phenotype of the accumulating DCs in the challenged mucosa, being CD14^+^CD11c^+^CD163^+^, resembles that of CD14^+^ monocytes in peripheral blood [16]. We therefore hypothesized that the rapid accumulation of CD14^+^CD11c^+^ DCs was caused by an increased recruitment of CD14^+^ monocytes from the circulation. Because the classical CD14^+^ monocytes, which constitute the vast majority of all monocytes in the circulation, can be identified by their expression of CCR2, we tested whether CD14^+^CD11c^+^ DCs in the challenged mucosa expressed this marker. In agreement with previous reports [17], [18] we showed that CCR2, as analyzed in two individuals, was highly expressed on most peripheral blood CD14^+^ monocytes, whereas only a fraction of myeloid DCs (CD14^−^CD11c^+^) expressed CCR2 (Figure 2A). Consistent with this we found that the majority of CD14^+^CD11c^+^ DCs in the intestinal mucosa also expressed CCR2; both in healthy controls, treated CD and active CD (Figures 2B and 3). In contrast, only a fraction of mucosal CD14^−^CD11c^+^ DCs and CD14^+^CD11c^−^ macrophages expressed this marker (Figures 2B and 3). Together, these findings further strengthen the notion that the CD14^+^CD11c^+^ DCs, which rapidly accumulate in the duodenal mucosa in response to gluten challenge, are recruited from circulating CD14^+^ monocytes. CD14^+^CD11c^+^ DCs also expressed CD209 (dendritic cell-specific intercellular adhesion molecule-3-grabbing non-integrin, DC-SIGN) both in healthy controls (n = 4; range 56–76%), in an untreated CD patient (77%), and a treated CD patient (73%) (Figure 2C). As previously reported [4] we found that DC-SIGN was highly expressed on macrophages, but virtually absent on CD14^−^CD11c^+^ DCs (Figure 2C).

### The increase of CD14^+^CD11c^+^ DCs precedes architectural changes and increase of CD3^+^ IELs

**Figure 4:**
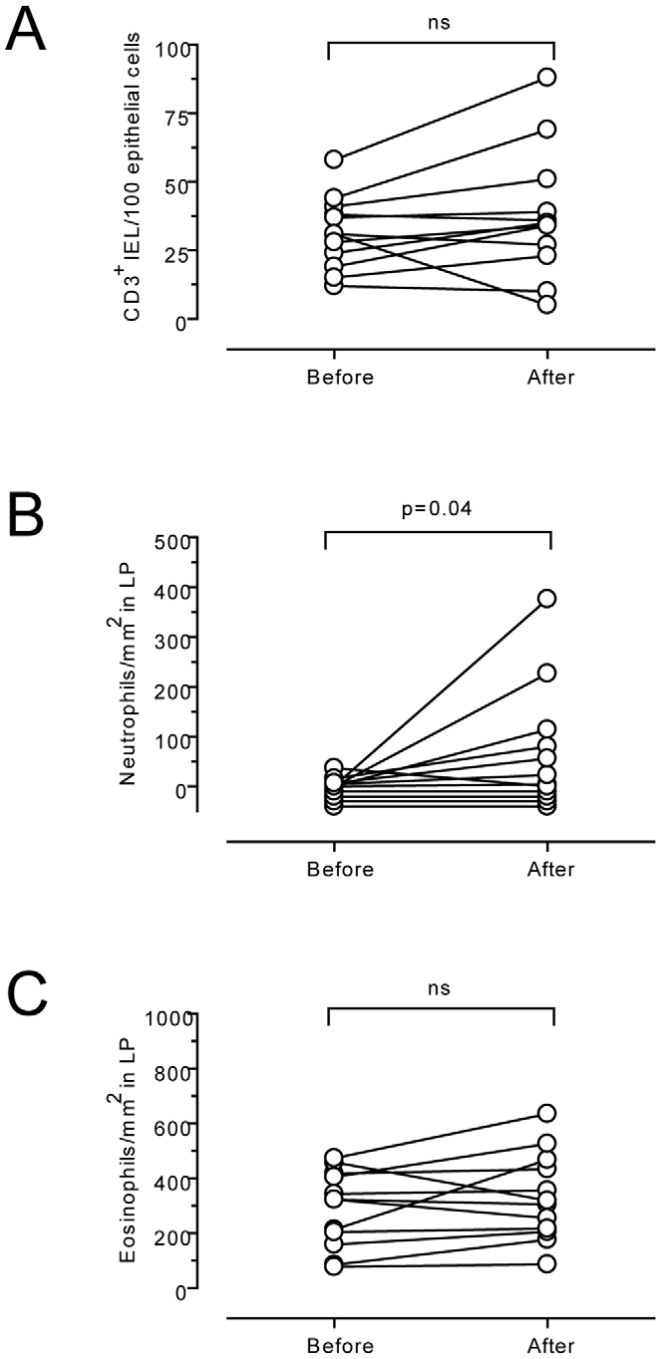
Neuthrophils are increased in duodenal mucosa of celiac disease patients after short-term gluten challenge. Density of CD3^+^ intraepithelial lymphocytes (IELs) per 100 epithelial cells (A); neutrophils per mm^2^ (B); and eosinophils per mm^2^ (C) in the lamina propria (LP) in sections of duodenal mucosa from celiac disease patients on gluten-free diet before and after a three-day gluten challenge. Paired data are connected by lines. ns = not significant.

The histopathology of the CD lesion is characterized by villous blunting, crypt hyperplasia and increased numbers of IELs [3]. Next we wished to determine whether accumulation of CD14^+^CD11c^+^ DCs preceded typical features of an established celiac lesion. In the same biopsy material, Brottveit et al. recently showed that in most patients there were no significant changes in tissue architecture after a three-day gluten challenge [6]. Only four of twelve patients had changes according to Marsh classification. As increased numbers of CD3^+^ IELs is one of the first signs of CD [19], [20], we counted CD3^+^ IELs in all samples. Importantly, no difference in the number of CD3^+^ IELs was detected comparing tissue obtained before and after challenge (Figure 4A). Thus, the increase of CD14^+^CD11c^+^ DCs precedes the histopathological hallmarks of an active CD lesion.

Increased recruitment of neutrophils and eosinophils are also features of the CD lesion [21], [22]. Of these immune cells only neutrophils showed a modest but significant increase (p = 0.04) after the short-term challenge (Figures 4B and 4C and Figure S1). Increased neutrophil recruitment demonstrated that the exposure to gluten triggered an inflammatory response.

### CD14^+^CD11c^+^ monocytes from blood efficiently present gluten to gluten-specific T-cell clones

**Figure 5:**
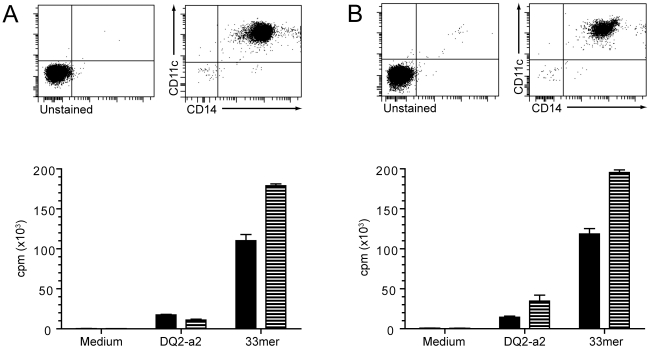
Peripheral blood CD14^+^ monocytes efficiently present gluten to gluten-specific T cell clones. Purity of CD14^+^CD11c^+^ monocytes isolated from peripheral blood mononuclear cells of two individuals are shown (A and B, upper panels). The monocytes were incubated with medium or two different gluten peptides ±100 U/ml IFN-γ for 24 hours, washed and incubated with a T-cell clone for 72 hours. The proliferative T-cell response (measured by thymindine-incorporation) is shown (A and B, lower panels). Experiments with IFN-γ are indicated (hatched columns).

We were not able to isolate CD14^+^CD11c^+^ DCs from the celiac lesion to test their antigen-presenting capability *in vitro*. Instead, we isolated CD14^+^CD11c^+^ monocytes from peripheral blood of two HLA-DQ2^+^ individuals and tested the ability of these cells to present gluten peptide antigen to an HLA-DQ2-restricted gluten-specific T-cell clone. From both individuals, the purified monocytes efficiently activated the T cells in an antigen dose-dependent manner. Preactivating the monocytes with IFN-γ further increased their antigen presenting capacity (Figure 5).

### No changes of cell densities in gluten-sensitive controls

**Figure 6:**
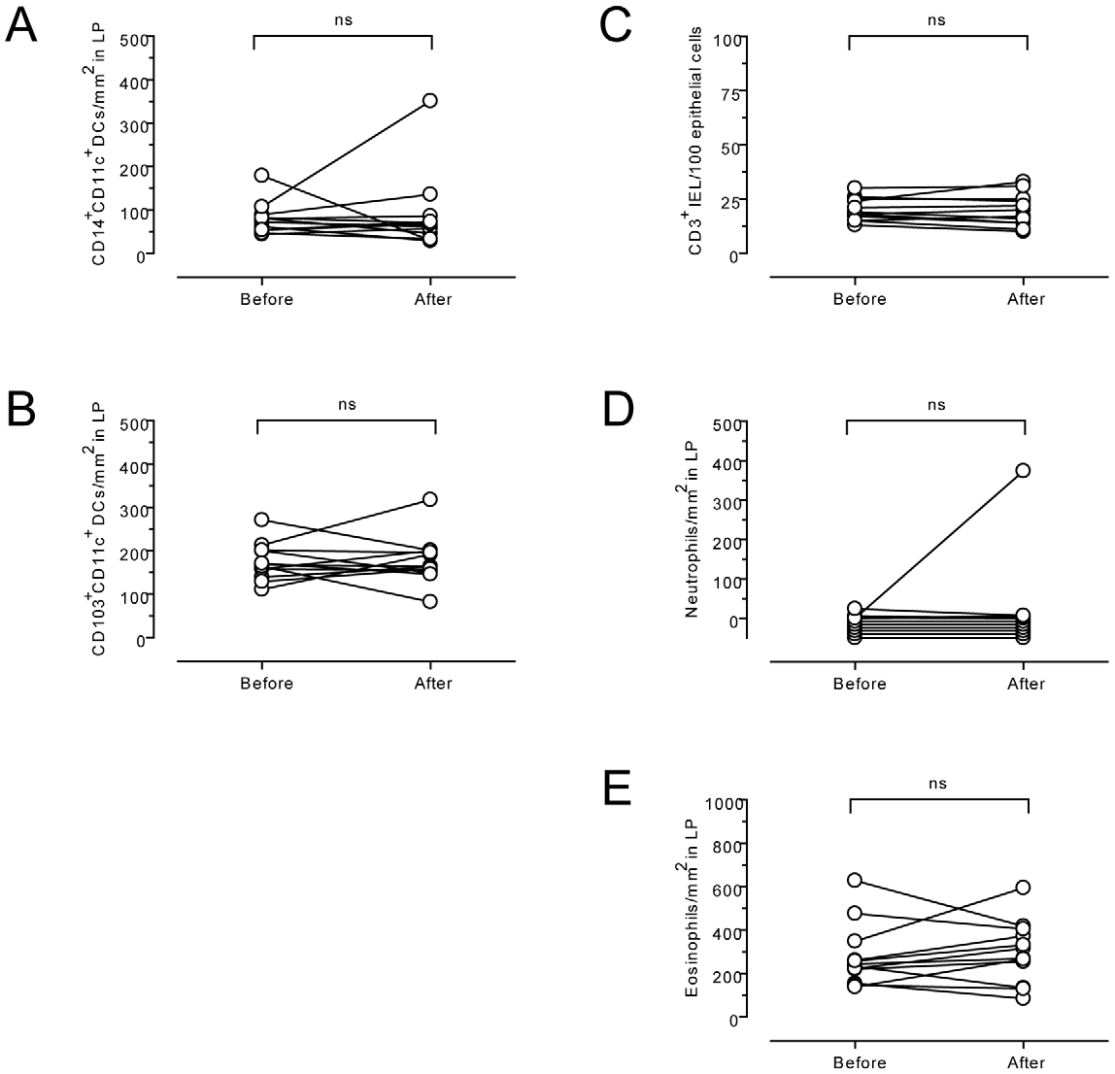
Density of various leukocyte subsets remains unchanged in duodenal mucosa of gluten-sensitive control subjects after short-term gluten challenge. Density of CD14^+^CD11c^+^ dendritic cells (DCs) per mm^2^ (A) and CD103^+^CD11c^+^ DCs per mm^2^ (B) in the lamina propria (LP); CD3^+^ intraepithelial lymphocytes (IELs) per 100 epithelial cells (C); neutrophils per mm^2^ (D) and eosinophils per mm^2^ (E) in the LP in sections of duodenal mucosa from gluten-sensitive control subjects on gluten-free diet before and after a three-day gluten challenge. Paired data are connected by lines. ns = not significant.

Next we wanted to examine whether the observed changes might represent an innate response to gluten independently of CD. To this end, we examined biopsy samples from gluten-sensitive control subjects on GFD who were challenged with gluten as described above. The gluten-sensitive controls were HLA-DQ2^+^ subjects without confirmed CD diagnosis, but with gluten-induced symptoms that subjectively improved on a GFD. As recently reported, no histopathological changes according to Marsh classification or tetramer-positive CD4^+^ T cells in peripheral blood were observed in this control group, although these patients experienced symptoms during the three-day gluten challenge [6]. Interestingly, as opposed to CD patients, no significant changes in the density of CD14^+^CD11c^+^ DCs, CD103^+^ DCs, neutrophils, eosinophils or CD3^+^ IELs were found after challenge (Figure 6). This finding indicates that the rapid recruitment of CD14^+^CD11c^+^ DCs and neutrophils, as well as the decrease of mucosal CD103^+^ DCs in response to gluten challenge are specific for CD.

## Discussion

We have studied changes in duodenal mucosal APC populations after a three-day gluten challenge in CD patients in remission and in gluten-sensitive control subjects. In the CD patients we found alterations in the APC populations in response to the challenge that preceded morphological changes in most participants. These changes resulted in a relative composition of APCs similar to that found in untreated CD [5] and were not observed in the gluten-sensitive controls. The findings suggest that the changes in the duodenal APC composition occur rapidly to gluten exposure and represent an early and integrated part of the immune reaction leading to CD.

The most pronounced difference was observed for the population of CD14^+^CD11c^+^ DCs. The density of these cells was increased almost three-fold in the celiac duodenal mucosa after the three-days gluten challenge, similar to that found in established celiac lesions [5]. Most notably, the increase preceded the typical architectural changes as well as an increase of IELs and eosinophils, which suggests that these cells may be important for disease development. There is a striking phenotypic resemblance between the DC subset that accumulated in the challenged mucosa and the classical monocytes in peripheral blood. Both cell populations express CD14, CD11c, CD163 and CCR2, which is a unique combination of markers in both blood and tissue. Although not formally demonstrated it is therefore tempting to speculate that the CD14^+^CD11c^+^ DCs accumulating in the tissue are derived from circulating CD14^+^ monocytes [5]. CD14^+^CD11c^−^ macrophages had lower expression of CCR2, suggesting that resident macrophages originating from monocytes might downregulate CCR2 [23].

An increasing body of evidence suggests that monocytes have the capacity to differentiate into efficient DCs in tissues. It was recently reported that in mice the expression of DC-SIGN/CD209 distinguishes monocyte-derived DCs (Mo-DCs) from classical DCs both in cell suspension and lymph nodes. Furthermore, these Mo-DCs (being CD14^+^CD11c^+^) were demonstrated to have strong antigen-presenting activity [24]. Most notably, we found that most CD14^+^CD11c^+^ DCs in the duodenal mucosa also express DC-SIGN/CD209, which is a putative marker of fully differentiated Mo-DCs. It is therefore conceivable that the recruited CD14^+^CCR2^+^ monocytes rapidly differentiate into efficient HLA-DQ^+^ APCs that activate gluten-reactive T cells residing in the intestinal mucosa. In agreement with this notion, we demonstrated that CD14^+^CD11c^+^ monocytes isolated from peripheral blood presented gluten peptides efficiently to gluten-specific T cells in a HLA-DQ-restricted manner. This is in line with our previous data showing that CD11c^+^ cells from duodenal biopsies are capable of presenting antigen to gluten specific T cells *in vitro* [4].

The gluten-sensitive subjects served as controls as they experienced gluten related symptoms, which we consider relevant, and as we were unable to recruit a control group of completely healthy subjects who adhered to a strict GFD. The mechanisms leading to symptoms in gluten intolerant patients are poorly understood. While integrated innate and adaptive immune responses appear important in the pathogenesis of CD [25], it is speculated whether symptoms of gluten intolerant patients may result from an unaccompanied innate immune response to gluten [26]. However, conflicting results are reported on the effect of gluten on the innate immune system. It has been reported that *in vitro* challenge of CD14^+^ human monocytes with digested gliadin causes maturation of the monocytes into DCs regardless of genetic predisposition or presence of CD in the cell donors [27], [28]. This gives credence to the above notion, but is at variance with the observation that stimulation of human duodenal biopsies with digested gliadin only gives activation of the innate immune system in CD patients and not in healthy controls [29]. Neutrophils and APCs both belong to the innate immune system, and in our study we observed early changes in these cell populations in the CD patients. In the established celiac lesion, the density of neutrophils and CD14^+^CD11c^+^ APCs are reported to increase [5], [21], [30]. Therefore, it was of interest to look at these cell populations in particular in gluten-sensitive control subjects before and after a short-term gluten challenge. The fact that the density of either APCs or neutrophils changed significantly in gluten-sensitive controls after challenge, demonstrates that the changes in these cell populations are restricted to CD. Moreover, the findings suggest that the symptoms reported in these patients upon the three-day gluten challenge, likely do not relate to innate immune activation by gluten in APCs or neutrophils.

The cues that lead to changes in the composition of the APC subpopulations in CD could possibly involve activation of T cells. Experiments *in vitro* have demonstrated that CD3^+^ T cells from duodenal biopsies of CD patients challenged with peptic-tryptic gluten digest or a gliadin fragment were activated when harvested after 24 hours as shown by upregulation of CD25 [29], [31]. Thus, it is to be expected that T cell activation takes place within the time frame of three days. Moreover, IFN-γ is shown to be produced both in biopsies and CD4^+^ T cells from CD patients upon 24 hours *in vitro* gluten challenge [32], [33]. Conceivably, in treated CD patients there might be interaction between primed T cells and APCs taking place at an early stage after gluten challenge. The observation that gluten exposure does not lead to changes in neutrophils and APC subpopulations in gluten-sensitive control subjects supports the model that activation of gluten specific T cells is implicated in the innate immune response in CD.

Taken together, we have found that CD14^+^CD11c^+^ DCs rapidly and selectively increase in the gluten challenged duodenal mucosa of treated CD patients. This did not occur in gluten-sensitive control subjects, making the gluten-induced recruitment of CD14^+^CD11c^+^ DCs specific for CD. Accumulation of this DC subset prior to induction of architectural changes and increase in IELs suggests that they are directly involved in the immunopathology of CD.

## Supporting Information

Figure S1 **Immunostaining of neutrophils in duodenal mucosa.** Immunoenzyme staining for neutrophil elastase to visualize neutrophils (arrows) on formalin-fixed and paraffin-embedded sections from duodenal mucosa of normal individuals and patients with untreated celiac disease. Original magnification X 400.
(TIF)
