# Supplementary figures and images for: Rapid Accumulation of CD14+CD11c+ Dendritic Cells in Gut Mucosa of Celiac Disease after in vivo Gluten Challenge
Source: PLoS One. 2012 Mar 16;7(3):e33556. doi: 10.1371/journal.pone.0033556 (PMC3306402; doi:10.1371/journal.pone.0033556)

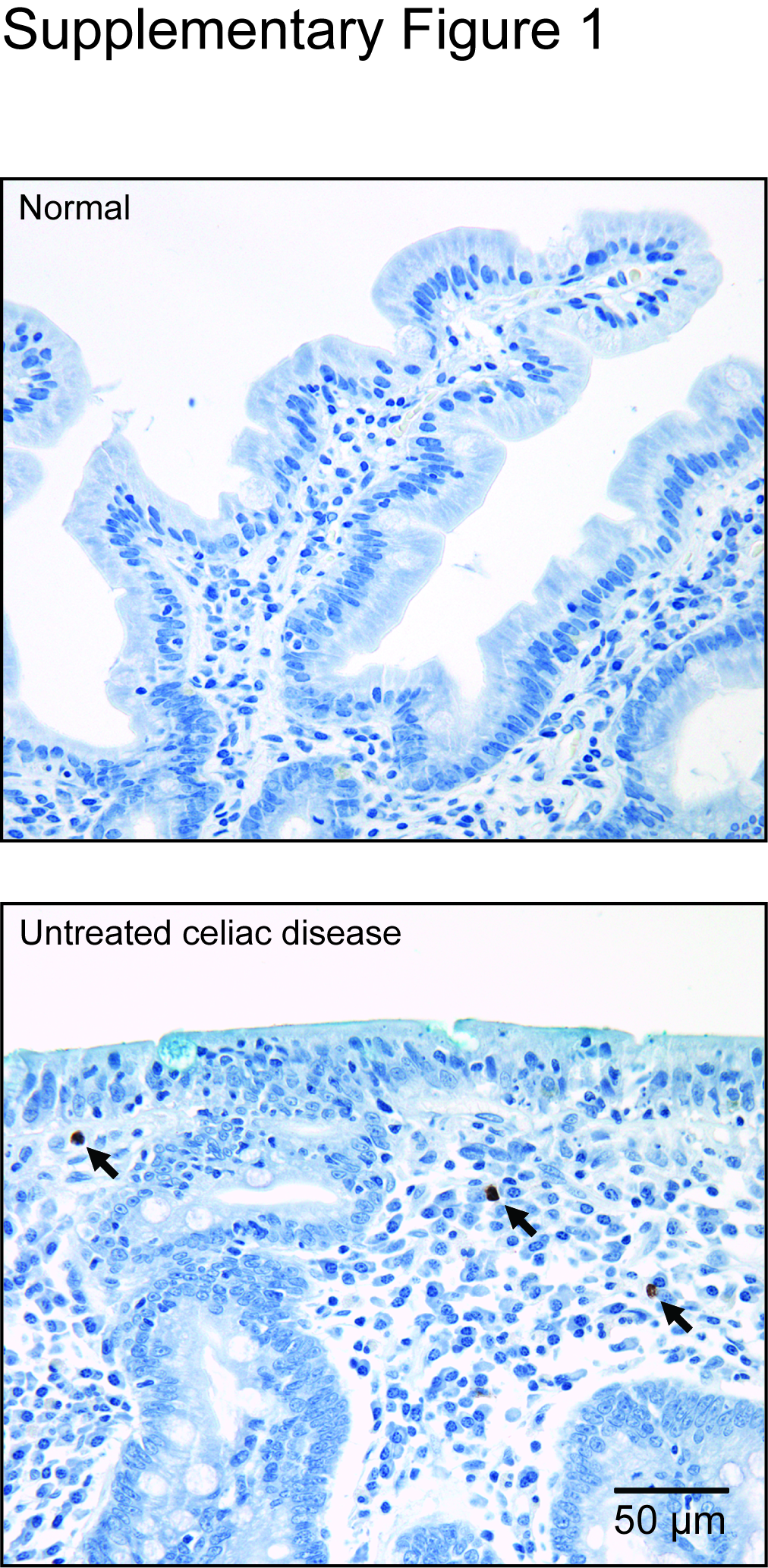

Supplement: Figure S1 — Immunostaining of neutrophils in duodenal mucosa. Immunoenzyme staining for neutrophil elastase to visualize neutrophils (arrows) on formalin-fixed and paraffin-embedded sections from duodenal mucosa of normal individuals and patients with untreated celiac disease. Original magnification X 400. (TIF) [file pone.0033556.s001.tif]
